# A Novel Four-Gene Signature as a Potential Prognostic Biomarker for Hepatocellular Carcinoma

**DOI:** 10.1155/2021/1452801

**Published:** 2021-12-14

**Authors:** Guangfeng Wang, Hao Zou, Yujie Feng, Wei Zhao, Kun Li, Kui Liu, Bingyuan Zhang, Chengzhan Zhu

**Affiliations:** Department of Hepatobiliary and Pancreatic Surgery, The Affiliated Hospital of Qingdao University, No. 16 Jiangsu Road, Qingdao 266003, China

## Abstract

Hepatocellular carcinoma (HCC) is a common malignant tumor with high incidence and mortality rates. However, a reliable prognostic signature has not yet been confirmed. Thus, the purpose of the present study was to develop a biomarker with high specificity and sensitivity for the diagnosis and prognosis of patients with HCC. The mRNA expression profiles of HCC were obtained from the GSE19665, GSE41804, and TCGA databases. Subsequently, 193 differentially expressed genes (DEGs) were identified from the intersection of the data from the three datasets. Bioinformatics analysis showed that the identified DEGs are related to the cell cycle, oocyte meiosis, and p53 signaling pathway, among other factors, in cancers. A protein-protein interaction (PPI) and a functional analysis were performed to investigate the biological function of the DEGs and obtain the candidate genes using the MCODE of Cytoscape. The candidate genes were introduced into the TCGA database for survival analysis, and the four candidate genes that were hub genes and meaningful for survival were retained for further verification. We validated the gene and protein expression and determined the prognosis of our patient cohort. In addition, we evaluated the biological functions regulating tumor cell proliferation and metastasis *in vitro*. According to the ROC curve analysis of gene expression in clinical samples, it was found that the four genes can be used to predict the diagnosis. A survival analysis based on data from the TCGA database and clinical samples showed that the four genes may be used as biomarkers for providing prognoses for patients. The cell functional experiments revealed that these four genes were related to tumor proliferation, migration, and invasion. In conclusion, the genes identified in the present study could be used as markers to diagnose and predict the prognosis of patients with HCC and guide targeted therapy.

## 1. Introduction

Hepatocellular carcinoma (HCC) is the fourth leading cause of cancer mortality worldwide and is one of the most common malignant cancers because of limited treatment options and poor prognosis [1]. The main treatment strategies include hepatectomy, liver transplantation, and targeted therapy [2, 3]. Because of microvascular invasion and heterogenicity [4, 5], early recurrence and metastasis after the surgery and poor responses to the targeted therapy are the main causes of short long-term survival [6]. Therefore, significant targets that could predict the prognosis of HCC and be the probable targets of therapy are urgently required.

Bioinformatics is widely used to comprehensively analyze the datasets with large numbers of cases to assess the genes related to the prognosis of liver cancer and/or to identify the genes that can be used as therapeutic targets. At present, most gene biomarkers are used to predict the prognosis and survival of cancer patients [7, 8] and provide guidance for further treatment decisions. For instance, Li et al. used bioinformatics to identify several key biomarkers that provide a candidate the diagnostic target and treatment for HCC [9]. It is different from the genes we screened for in the present study. Similarly, the previous research has only used the TCGA database, however, these results are different from the results presented in the present study [10]. Furthermore, in the previous bioinformatics analyses, there were few functional experiments to verify the results, and we have included this in the present study.

In the present study, the datasets of the expression profiles were downloaded from the GEO and TCGA databases to obtain the DEGs. Bioinformatic functional analyses were conducted to identify the prognosis-related genes and cancer-related molecular mechanisms. A new signature has been identified as a prognostic biomarker for HCC. The biological functions of the hub genes were experimentally confirmed.

## 2. Materials and Methods

### 2.1. Datasets and DEGs Identification

Two datasets (GSE41804 and GSE19665) of mRNA gene expression were downloaded from the GEO database (https://www.ncbi.nlm.nih.gov/geo/). The gene expression profiles were downloaded from the TCGA database (https://cancergenome.nih.gov/). The GSE41804 dataset contains the paired samples of 20 HCC tissues and 20 adjacent tissues from 20 patients. The GSE19665 database contains 10 HCC and 10 non-HCC samples from 10 patients. We also obtained 371 tumor and 50 nontumor samples from the TCGA database for validation purposes.

In the GEO database, GEO2R is a convenient online tool for users to compare the datasets in a GEO series to distinguish the DEGs between the HCC and noncancerous samples. The*p*-values and the Benjamini–Hochberg test were used to coordinate the significance of the DEGs obtained and reduce the number of false positives. Subsequently, the DEGs were screened against the corresponding datasets based on a *p*-value < 0.05, and |logFC| (fold change) ≥ 2 was used as a threshold to improve the credibility of the results. Then, the lncRNAs and miRNAs obtained from the TCGA database were eliminated. We acquired three groups of mRNA expression profiles after processing the data. The applet (http://bioinformatics.psb.ugent.be/webtools/Venn/) was used to determine which data in the three groups intersect.

### 2.2. PPI Network Construction

The PPI network was predicted using the Search Tool for the Retrieval of Interacting Genes (STRING; http://string-db.org) online database [11]. Research on the functional interactions between the proteins can provide a better understanding of the potential mechanisms underlying the occurrence or development of cancers. In the present study, the PPI interactions with a combined score >0.4 were regarded as statistically significant in the STRING database and were extracted to build a network of DEGs. We then visualized the molecular interaction network using Cytoscape (Version 3.6.1), which is an open-source bioinformatics software [12]. The Molecular Complex Detection (MCODE) [13] is a plug-in packaged with the Cytoscape software. The main function of MCODE is to cluster and construct the functional modules by means of topology in a huge gene (protein) network to find dense rendezvous spots. MCODE scores > 5, node density cutoff = 0.1, degree cutoff and K-core = 2, node score cutoff = 0.2, and a maximum depth of 100 were used as the benchmarks for the gene module selection.

### 2.3. GO and KEGG Pathway Enrichment Analyses

The cluster profiler package [14] obtained from Bioconductor (http://bioconductor.org/) is a free online bioinformatics package in R. It contains biological data and analysis tools that provide a systematic and comprehensive biological functional annotation information of the large-scale genes or proteins that help the users extract biological information from them. Gene Ontology (GO) enrichment analysis is widely used for gene annotation and the analysis of the biological processes of DEGs [15]. Statistical significance was set at *p* < 0.05. A KEGG pathway enrichment analysis (http://www.genome.jp/kegg/pathway.html) provides an understanding of the advanced functions of the biological systems at the molecular level. It is widely used for large-scale molecular datasets produced by high-throughput experimental technologies [16].

### 2.4. Survival Analysis and Expression Levels of the Hub Genes

The survival analysis of the hub genes was performed using Kaplan–Meier analysis. Using GEPIA (http://gepia2.cancer-pku.cn), a TCGA visualization website, all of the expression information of the patients with HCC in the TCGA database were divided into high- and low-expression groups according to the median of each gene expression level. In addition, the gene expression of patients in our hospital was obtained using real-time PCR, and the corresponding survival analysis was performed according to the aforementioned method of analysis. Furthermore, the box plots of GEPIA were plotted to reflect the expression levels of each gene.

### 2.5. Establishment and Validation of the Prediction of the Signature

The signature was applied to a cohort of patients with HCC in our hospital to verify its ability to predict HCC. The expression of the genes in patients with HCC was measured, and the ROC curve was obtained using GraphPad Prism 7.

### 2.6. Cox Regression Analysis and Prognostic Validation of the Signature

The intersection of the DEGs among the three cohorts of mRNA expression profiles was selected to construct the predictive character for survival. The aforementioned hub genes in the TCGA cohort were incorporated into a multivariate Cox regression model using the online Kaplan–Meier plotter [17] to obtain the survival analysis and verification of the biomarkers. The prognosis risk score for predicting the overall survival (OS) of HCC patients was determined by multiplying the expression level of these genes (exp) by a regression coefficient (*β*) obtained from the multivariate Cox regression model. The algorithm used was Risk score=EXP_gene_1__*∗β*_gene_1__+EXP_gene_2__*∗β*_2gene_2__+⋯EXP_gene*n*_*∗β*_gene_*n*__. A total of 364 HCC patients with accessible data were selected for the individual survival analyses. The median risk score was treated as a threshold. Based on this, the HCC patients were divided into low-risk and high-risk groups. Similarly, the aforementioned algorithm was applied to a cohort of HCC patients from our hospital to verify the prognostic value of the hub genes.

### 2.7. Cell Culture and Plasmid Transfection

The human HCC cell line M3 was obtained from the Shanghai Institute for Biological Sciences, Chinese Academy of Sciences. The cell line was cultured in HyClone Dulbecco's Modified Eagle's Medium (DMEM) mixed with 10% fetal bovine serum (FBS) and 1% penicillin and streptomycin in a humidified incubator (Thermo Scientific, USA) at 37°C and 5% CO_2_. The control and gene-overexpressing plasmids were purchased from Genechem Co. Ltd (Shanghai, China). EXO1, CYP2C8, and CLEC1B share the GV141 vector, which contains a multiple cloning site followed by a 3FLAG tag downstream of the CMV promoter, as well as the neomycin gene downstream of the SV40 promoter. In addition, the vector of GYS2 is GV230, which contains a multiple cloning site followed by EGFP downstream of the CMV promoter, as well as the neomycin gene downstream of the SV40 promoter. Transient transfection was performed using Lipofectamine 2000 (Invitrogen, USA) according to the manufacturer's instructions.

### 2.8. Wound Healing Assay

The cells were cultured in a six-well cell culture plate overnight at 37°C and 5% CO_2_. The following day, transient transfection was performed, and the cells were cultured for 24 h. The cells were washed twice with phosphate-buffered saline (PBS). A 200 *μ*L tip of a pipette was then used to make cross scratches at the bottom of the wells. The images were captured at 0 and 24 h using a microscope (Nikon, Japan).

### 2.9. Cell Migration and Invasion Assays

The migration assay (Transwell assay) was conducted after transfection and culture. The cells of the control and experimental groups were counted and placed in the transwell chambers. The HCC cells (105 cells) were fully mixed with the serum-free DMEM and added to the interior of the chambers. Then, 600 *μ*L of DMEM with 10% FBS was added to the bottom of the 24-well cell culture plate. The chambers were placed in the plate wells. Matrigel was added to the chambers for invasion assay. During culturing in the incubator for 10 h to 24 h, methanol was added to the chambers to fix the cells that had migrated or invaded. At the end of the treatment, the cells were stained with crystal violet, observed, and counted under a microscope.

### 2.10. Cell Growth and Cloning Assays

The transfected cells were incubated in a 96-well cell culture plate overnight at 37°C and 5% CO_2_ to attach CYP2C8: 5000 cells/well, EXO1, CLEC1B, and GYS2: 2000 cells/well. A Cell Counting Kit-8 (CCK8) was used to detect the viability of the cells at different points in time. The cells were cultivated for 2 h in an incubator after the addition of 100 *μ*L DMEM with 10 *μ*L CCK-8 reagent per well. Subsequently, the absorbance optical density (OD) was measured at 450 nm. For the cloning assay, 1000 transfected cells per well were implanted in a six-well culture plate and cultured for 14 days at 37°C and 5% CO_2_. The cells were fixed with 10% formalin, stained with crystal violet, and counted.

### 2.11. HCC Tissue Samples

We ethically collected a total of 40 pairs of tumor and paratumor tissues from the patients who underwent hepatectomy between 2016 and 2019 at the Affiliated Hospital of Qingdao University. All patients were confirmed to have HCC by postoperative pathology, and their clinical and follow-up data were integrated into the present study. The present study complied with the Declaration of Helsinki, and all sample collections were approved by the Ethics Committee of The Affiliated Hospital of Qingdao University. Informed consent was obtained from each patient. None of the tissue samples was exposed to any preoperative radiotherapy or chemotherapy. Paired cancer and paracancerous tissues were collected in RNA protective additive-filled microtubes and immediately frozen in a medical refrigerator at −80°C.

### 2.12. Real-Time PCR Analysis for Verification of the Signature

Total RNA was extracted from the collected tumor and nontumor tissue samples using RNAiso Plus (total RNA extraction reagent, Takara Inc. Japan) according to the manufacturer's instructions. Subsequently, the fineness of the total RNA was estimated using the A260/A280 ratio, and the corresponding DNA was obtained using the PrimeScript™ RT reagent Kit (Takara Inc. Japan). Quantitative real-time PCR (qRT-PCR) was performed using a LightCycler 480 (Roche, Basel, Switzerland) and Takara TB Green™ Premix Ex Taq™ II (Tli RNaseH Plus, Takara Inc. Japan). The thermocycling procedure was as follows: preincubation at 95°C for 5 min, followed by 35 temperature cycles (each involving denaturation at 95°C for 20 s, annealing at 60°C for 30 s, and extension at 72°C for 20 s). The procedure was repeated 3 times. An analysis of the melting curve was performed on each sample to verify amplification specificity. The primer sequences of nine genes are shown in Table 1. The data were standardized using a control group of *β*-actin.

### 2.13. Statistical Analysis

Data analyses were performed using GraphPad Prism software (version 7.0; San Diego, CA, USA). All experimental data are expressed as mean ± SD. The statistical significance of the results was analyzed by the one-way analysis of variance (ANOVA) for comparisons between three or more groups and the Student's *t*-tests for comparisons between the two groups. The differences were considered statistically significant at *p* < 0.05. All experiments were performed in triplicate.

## 3. Results

### 3.1. DEGs Identification and Identification of the Hub Genes

**Figure 1:**
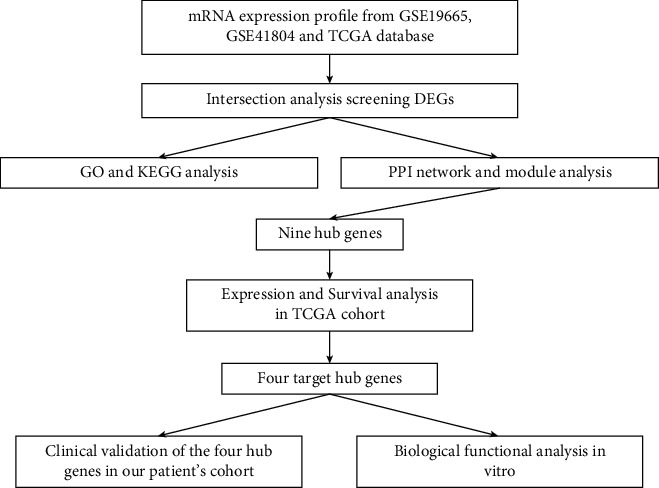
Workflow of this study to construct a four-gene signature in HCC.

**Figure 2:**
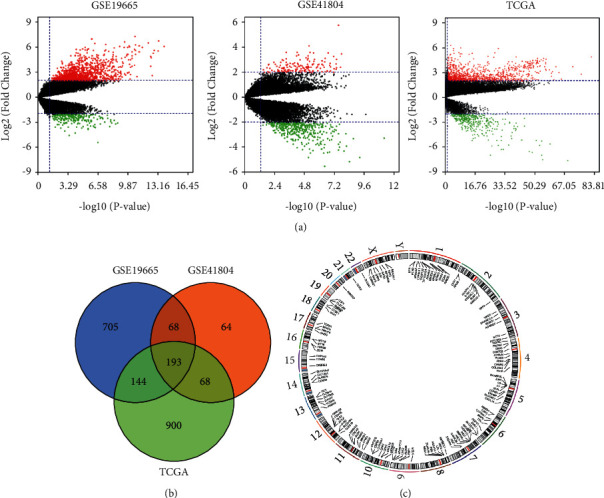
DEGs in HCC. (a) Volcano plot of all genes expression profiles in GSE16515, GSE28735, and TCGA. The red represents the mRNA with high expression level, while the green represents the mRNA with low expression level. (b) Venn diagram showing DEGs in GSE16515, GSE28735, and TCGA. (c) Circos plots showing the position of DEGs on the chromosome.

The entire research process is illustrated in Figure 1. After processing the GSE19665, GSE41804, and TCGA data, the DEGs were identified between HCC and nontumor tissue using *p* < 0.05 and |logFC| ≥ 2 as the thresholds. There were 1341, 155, and 943 upregulated genes in GSE19665, GSE41804, and TCGA, respectively. There were 224, 389, and 362 downregulated genes in GSE19665, GSE41804, and TCGA, respectively. The up- and downregulated genes are shown with a volcano plot in Figure 2(a). One hundred and ninety-three DEGs were identified by the intersection of the genes between the three cohorts (Figure 2(b)). The detailed positions on the chromosomes of these 193 genes are shown in the Circos plot (Figure 2(c)).

**Figure 3:**
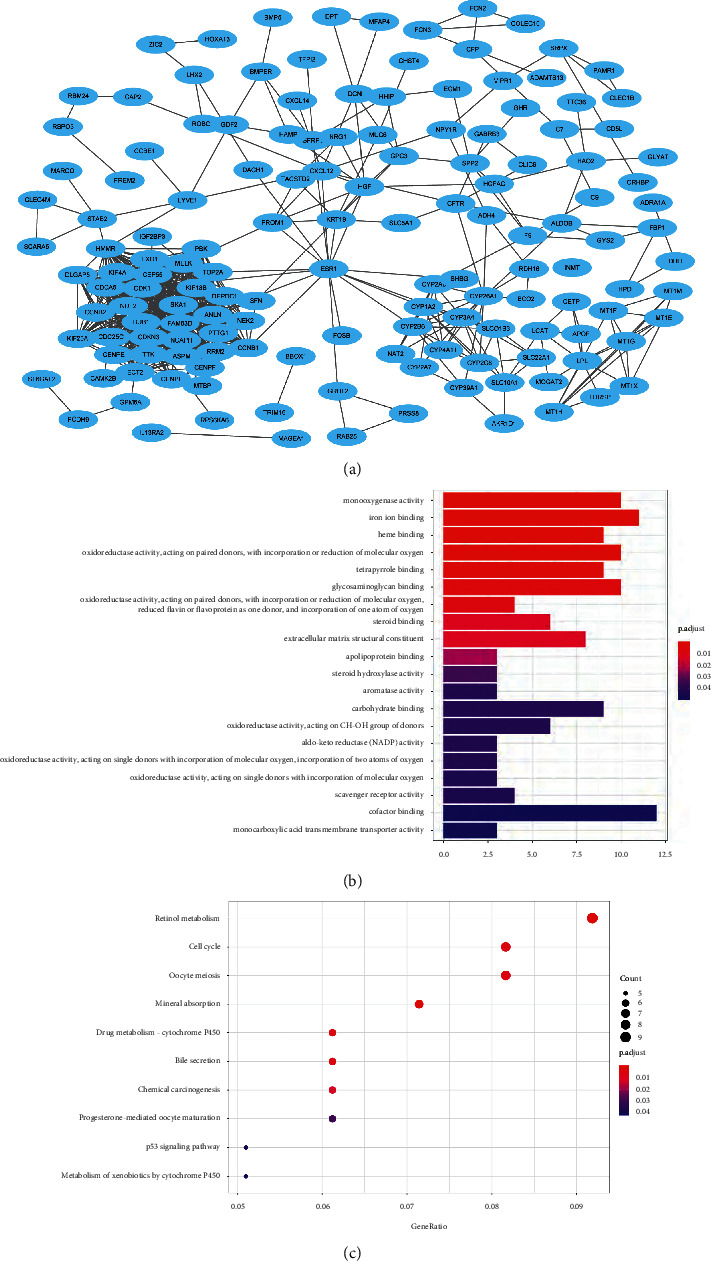
Function analysis of the DEGs. (a) PPI network of DEGs. (b) GO function analysis of the DEGs. (c) KEGG pathway enrichment of DEGs.

A PPI dataset was obtained from STRING and used to construct a PPI network of the DEGs. Subsequently, an interaction analysis was performed to visualize the interaction network using Cytoscape (Figure 3(a)). The results showed that MT1M, CYP2C8, CFP, EXO1, CLEC1B, GRHL2, SLCO1B3, HAMP, and GYS2 were the nine most highly ranked genes (Table 2).

### 3.2. Functional Enrichment and Survival Analysis of the Hub Genes

The GO enrichment analysis was conducted to investigate the biological functions, which indicated that the cellular processes and biological regulation were significantly enriched in the biological processes (BP). The main enrichments included the binding of iron ions, activity of monooxygenase, heme binding, and oxidoreductase activity (Figure 3(b)). KEGG pathway analysis showed that these genes were significantly enriched in the pathways related to retinol metabolism, the cell cycle, oocyte meiosis, and the p53 signaling pathway in cancers (Figure 3(c)). These results suggest that these genes are important for the pathogenesis and progression of HCC.

**Figure 4:**
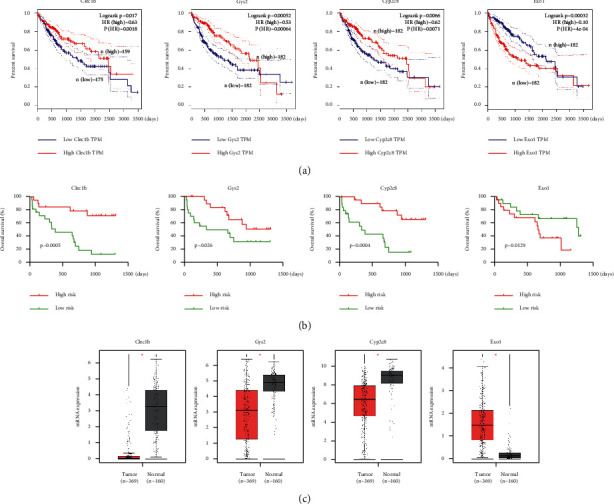
The situation of signatures in the TCGA database. (a) Kaplan–Meier survival plots of four genes in TCGA cohort. (b) Kaplan–Meier survival plots of four genes in our patient cohort. (c) Box plots showing the expression of four genes in TCGA cohort.

**Figure 5:**
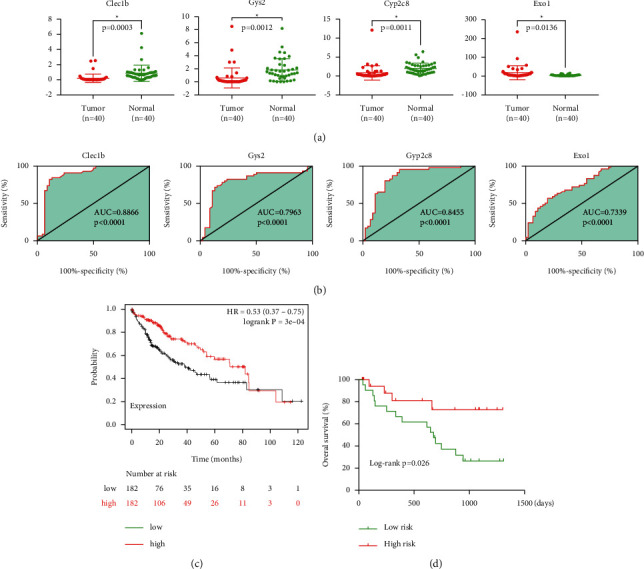
The validation of signatures in our patient cohort. (a) qRT-PCR analysis: mRNA expression levels of four genes in HCC and paired nontumorous liver tissues. (b) ROC analysis of four genes. (c) Kaplan–Meier survival plots of the four-gene signature in TCGA cohort. (d) Kaplan–Meier survival plots of the four-gene signature in our patient cohort.

A Kaplan–Meier analysis was performed to screen out the genes in the TCGA database that were related to overall survival (OS). Four of the nine genes were significantly correlated with the prognosis. The patients with a high expression level of CLEC1B (*p*=0.017), GYS2 (*p*=0.00052), and CYP2C8 (*p*=0.0066) and a low expression level of EXO1 (*p*=0.00032) had a favorable prognosis (Figure 4(a)). Then, we validated the function of predicting the prognosis of patients in our cohort using Kaplan–Meier analysis as shown in Figure 4(b). We then investigated the level of gene expression in the TCGA database (Figure 4(c)) and in our cohort (*n* = 40, *p* < 0.05, Figure 5(a)). Clinicopathological information is listed in Table 3. CYP2C8, CLEC1B, and GYS2 were downregulated, whereas EXO1 was upregulated in HCC (*p* < 0.05).

### 3.3. Predictive and Prognostic Indication of the Four-Gene Signature

To evaluate the four-gene signature for predicting HCC, an analysis of the ROC curve of each gene was performed according to the expression of each gene. As presented in Figure 5(b), the AUCs of EXO1, CLEC1B, GYS2, and CYP2C8 were 0.7339, 0.8866, 0.7963, and 0.8455, respectively.

To validate the function as a prognostic indicator, we divided the 364 patients in the TCGA database into low-risk (182 patients) and high-risk cohorts (182 patients) according to their median risk score. The OS of the high-risk cohort was superior to that of the low-risk cohort (log-rank *p*=0.0003, Figure 5(c)). A similar result was obtained for our cohort (log-rank *p*=0.026) with further validation, which indicated that the signature could be a favorable predictive biomarker of HCC (Figure 5(d)).

### 3.4. Biological Functions of the Four Hub Genes

**Figure 6:**
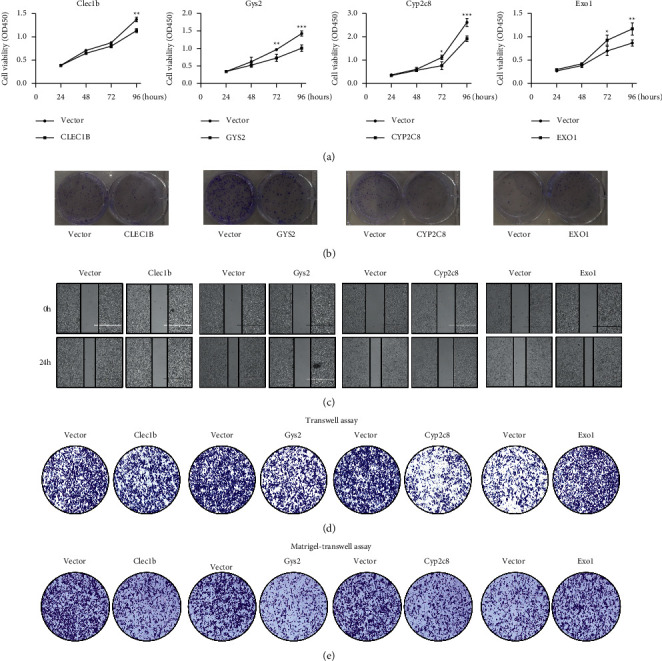
Biological function of gene overexpression of M3 cells. (a) Cell proliferation curves of the vector group and gene-overexpression group. (b) Colony assays of the vector group and gene-overexpression group. (c) Wound healing assays verifying the migration ability of M3 cells in vector group and transfected group. (d) Transwell assays validating the motility of vector group and gene-overexpression group. (e) Matrigel-transwell assays contrasting the invasiveness between the vector group and gene-overexpression group.

To investigate the biological functions of the genes, we overexpressed the genes by plasmid transfection in the M3 HCC cell line. The CCK8 and cloning assays indicated that the overexpression of CLEC1B, GYS2, and CYP2C8 inhibited cell proliferation, while EXO1 significantly promoted M3 cell proliferation (Figures 6(a) and 6(b)). The wound healing assay and Transwell assay demonstrated that the upregulation of CLEC1B, GYS2, and CYP2C8 restrained migration, while EXO1 accelerated the migration of M3 cells (Figures 6(c) and 6(d)). In addition, the Transwell assay with Matrigel showed that the overexpression of CLEC1B, GYS2, and CYP2C8 inhibited cell invasion, while the upregulation of EXO1 promoted the invasion of M3 cells (Figure 6(e)).

## 4. Discussion

Bioinformatics analysis is a useful tool for exploring the prognostic indicators of cancer and possible therapeutic targets. We performed an integrated analysis of two GEO datasets and the TCGA database and identified four hub genes based on a biofunctional analysis. A signature was established and validated to predict the prognosis of patients with HCC. In addition, the biological functions of these four genes were investigated experimentally. In the present study, the genes expressed in the nontumor liver tissues and tumor liver tissues from the two GEO and TCGA databases were analyzed and filtered to identify a novel set of four-gene signatures (CLEC1B, GYS2, CYP2C8, and EXO1) to diagnose and determine the prognosis of patients with HCC. CLEC1B, GYS2, and CYP2C8 were identified as tumor suppressors for the prognosis, however, EXO1 was determined to be an oncogene. In spite of GYS2 [18] and CYP2C8 [19], which are regarded as one hub gene with two signatures, their screening criterion was |logFC| > 1, while |logFC| ≥ 2 was adopted in our study. CLEC1B was identified as a new biomarker [20], however, because of a lack of verification of the survival model, its reliability remains low. Here, we used more stringent screening criteria and analyzed these four genes together to establish a survival model. The performance of the Kaplan–Meier analysis obtained from the TCGA information on the HCC patients demonstrated that the four-gene signature was a sensitive potential biomarker for predicting the prognosis of patients with HCC not only for each individual gene but also for the four-gene association. Synchronously, it is different from most other simple bioinformatics studies that use only one dataset [21, 22]. Furthermore, the clinical tissue data were analyzed using ROC to diagnose HCC. The ROC analysis consequently proved the accuracy and specificity of the four-gene signature in the diagnosis of HCC. The functional verification of these genes has seldom been conducted in other studies. Other studies remained theoretical. We also investigated the functions of the four genes corresponding to the signature at the cellular level and the level of expression of the corresponding proteins in the cancer and paracancerous tissues. In summary, a multidimensional analysis of these four genes firmly demonstrated that the combination of these four genes could effectively predict the prognosis of HCC patients.

Glycogen synthase 2 (GYS2) is a key enzyme in glycogen biosynthesis. GYS2 was significantly downregulated in HCC with glycogen loss, resulting in a poor prognosis. GYS2 inhibited tumor growth in HBV-related HCC by negative feedback in the p53 signaling pathway [23]. By a series of *in vitro* experiments, we confirmed that the overexpression of GYS2 can lead to the proliferation, metastasis, and invasion of HCC cells.

Exonuclease 1 (EXO1) is an exonuclease from the 5′ to 3′ end that participates in the regulation of the cell cycle checkpoint, the maintenance of replication forks, and the postreplication repair of DNA [24]. A deficiency in restarting the DNA replication pathway may lead to double-strand breaks, cell cycle arrest, cell death, or transformation, which may lead to cancer [25], and EXO1 is involved in this process. Therefore, the variations in EXO1 have been linked to various types of cancers [26]. Furthermore, several lines of previous research have reported a negative relationship between the overexpression of EXO1 and the prognosis of various cancers [27–30]. It has been reported that the overexpression of EXO1 leads to a poor prognosis in patients with HCC [31]. In addition, it has been shown that the overexpression of EXO1 is associated with a poor prognosis in breast cancer [32].

CYP2C8 is located in the cytochrome P450 gene cluster on chromosome 10q24 and can metabolize approximately 30% of the body's clinical drugs and various chemicals from the environment [33]. Moreover, KEGG analysis showed that CYP2C8 is related to retinol and chemical metabolism. A previous study pointed out that the OS of patients with HCC with low CYP2C8 was worse than that for those with high CYP2C8 [34, 35]. In addition, the low expression level of CYP2C8 was related to advanced clinicopathological features, including tumor stage and intrahepatic metastasis. According to the database, CYP2C8 is well-expressed in normal human livers, and CYP2C8 metabolizes paclitaxel [36]. Paclitaxel is prescribed in combination with the cytochrome P450 inhibitors to enhance its anticancer effects against various malignant tumors [37]. Therefore, this finding may explain why paclitaxel has effective antitumor activity *in vitro* but has no significant clinical effect on patients with HCC. Our research has further promoted the use of paclitaxel in patients with clinical liver cancer *in vitro*.

CLEC1B, a member of the C-type lectin domain family 1, is mainly related to the thromboses caused by platelet aggregation, platelet-mediated tumor proliferation, and metastasis [38, 39]. Furthermore, it has been previously reported that CLEC2 is significantly downregulated in the HCC tissues [40], which agrees with our results. A recent study also revealed that the downregulation of CLEC2 is related to the depth to which the tumor has invaded, lymph node metastasis, and the 5-year survival rate [41]. In the present study, we confirmed the role of CLEC1B, as reported by previous studies, that the overexpression of CLEC1B distinctly suppressed the proliferation, metastasis, and invasion of the HCC cells. We also confirmed that CLEC1B is a marker gene highly related to the progression of HCC and the low expression level of CLEC1B may be a significant prognostic factor, suggesting a poor clinical outcome. Furthermore, it can be used as a target for immunotherapy, which is consistent with the views of Hu et al. [42].

We believe that the signature of the four genes combined is a promising prognostic indicator for patients with HCC. However, there are some limitations to the present study. Firstly, the mechanism of gene regulation in HCC progression needs further investigation. Secondly, because of the shortage of clinical specimens and short-term follow-up, the data needs further validation with more patients [43].

## 5. Conclusion

Based on the results of the present study, which involved an analysis of the data in the databases and the experimental verification of the functions of genes, we identified a reliable signature to predict the prognosis of patients with HCC. In addition, the signature can be used as a guide for targeted cancer therapy. However, further studies are needed to develop the prognostic and diagnostic signatures for HCC.

## Figures and Tables

**Table 1:** mRNA PCR primer.

Gene name	Primer sequence

EXO1	F: TCGGATCTCCTAGCTTTTGGCTG
	R: AGCTGTCTGCACATTCCTAGCC

CYP2C8	F: GAGACAACAAGCACCACTCTGAG
	R: CAGTGTAAGGCATGTGGCTCCT

CLEC1B	F: TGGTGGCGTGTGATGGCTTTGA
	R: CACCTTGTAGGTAATTGCGCTGC

GYS2	F: CCAGTGACCACGCACAACATGA
	R: GTAAGGGACTGGTGGAGGATAG

*β*-actin	F: CCTCTCCCAAGTCCACACAG
	R: GGGCACGAAGGCTCATCATT

**Table 2:** List of 9 hub genes identified in the PPI network.

Gene symbol	Description

MT1M	Metallothionein 1M
CYP2C8	Cytochrome P450 family 2 subfamily C member 8
CFP	Complement factor properdin
EXO1	Exonuclease 1
CLEC1B	C-Type lectin domain family 1 member B
GRHL2	Grainyhead like transcription factor 2
SLCO1B3	Solute carrier organic anion transporter family member 1B3
HAMP	Hepcidin antimicrobial peptide
GYS2	Glycogen synthase 2

PPI, protein–protein interaction.

**Table 3:** The correlation of HCC clinic pathological variables with gene expression level in tissue samples.

Clinic pathological features	Low risk (*n* = 20)	High risk (*n* = 20)	*p*-value

Age (years)			
<60	13	8	0.113
≥60	7	12	
Gender			
Male	15	13	0.49
Female	5	7	
Smoking			
Yes	14	7	0.027
No	6	13	
Alcohol			
Yes	16	8	0.01
No	4	12	
AFP level (ng/L)			
≤400	8	16	0.01
>400	12	4	
Microvascular invasion			
Yes	13	6	0.027
No	7	14	
TNM stage			
I-II	8	6	0.507
III-IV	12	14	

HCC, hepatocellular carcinoma; TNM, tumor, lymph node and metastasis.

## Data Availability

The datasets used in this study are available from the corresponding author on reasonable request.
